# High expression of phosphorylated focal adhesion kinase predicts a poor prognosis in human colorectal cancer

**DOI:** 10.3389/fphar.2022.989999

**Published:** 2022-09-13

**Authors:** Guanyu Yu, Mengnan Xu, Leqi Zhou, Kuo Zheng, Xiaoming Zhu, Jinke Sui, Cheng Xin, Wenjun Chang, Wei Zhang, Fuao Cao

**Affiliations:** ^1^ Department of Colorectal Surgery, Changhai Hospital, Naval Medical University, Shanghai, China; ^2^ Department of Environmental Health, Naval Medical University, Shanghai, China

**Keywords:** p-FAK, colorectal cancer, prognosis, chemotherapy, immunohistochemistry

## Abstract

**Background:** Phosphorylated Focal adhesion kinase (FAK) has been reported to be intimately involved in various malignant tumors. The effect of p-FAK on colorectal cancer (CRC) is still disputable. The purpose of this study is to investigate the role of p-FAK in the prognosis of colorectal cancer.

**Methods:** The clinical significance of p-FAK expression in CRC was evaluated by immunohistochemistry in a large cohort, including carcinoma and para-carcinoma tissues from 908 patients, and normal tissues, adenoma, and metastasis tissues. The correlation between p-FAK expression and CRC occurrence was investigated in tumor and other tissues. Factors contributing to prognosis were evaluated using Kaplan-Meier survival analysis and Cox regression model.

**Results:** p-FAK is apparently overexpressed in CRC and metastasis tissues. Compared with low p-FAK expression, patients with high p-FAK expression had shorter overall survival [hazard ratio (HR), 2.200; 95% confidence interval (CI), 1.265–3.452; *p* < 0.01] and disease-free survival (HR, 2.004; 95% CI 1.262–3.382; *p* < 0.01) in multivariate Cox analysis after adjusting other prognostic factors. High p-FAK expression was also related to a worse chemotherapeutic response in patients who achieved adjuvant chemotherapy (*p* < 0.01).

**Conclusion:** Expression level of p-FAK is an independent risk factor and can serve as a prognostic biomarker for CRC. High p-FAK expression predicts an unfavorable prognosis of CRC as well as poor chemotherapeutic response.

## Introduction

Colorectal cancer (CRC) is the third most common cancer, accounting for approximately 10% for of all annually cancer-related mortality worldwide (Bray et al., 2018). In China, it is estimated that CRC will become the fifth most common cause of cancer-related death both in men and women with 590,000 newly diagnosed cases and 180,000 deaths in 2022 (Xia et al., 2022). Advances in pathogenesis and pathological understanding, surgical techniques, and diversity of treatment methods have provided additional options for primary and metastatic CRC patients. Although these modified regimens lead to a two-fold increase in survival up to 3 years, the best survival occurs in those patients without recurrence or metastasis (Dekker et al., 2019). The managements have limited impact on cure-rate and the response of CRC patients to these therapeutic strategies is quite heterogenous. Nearly 25% of patients with localized lesion will develop metastases and 10%–30% of patients will develop recurrence after treatment intervention, and many of whom will die from that (Buccafusca et al., 2019; Biller and Schrag, 2021).

So far, cancer heterogeneity, prognostic prediction and selection of systemic therapies relies on histological diagnosis and the tumor-node-metastasis (TNM) staging system defined by the American Joint Committee on Cancer/Union for International Cancer Control (AJCC/UICC) (Nagtegaal et al., 2011). However, even within the same stage, the clinical outcomes and drug responsiveness of CRC patients can be significantly heterogeneous (Dekker et al., 2019). The high recurrence and metastases rate of CRC and outcome prediction after treatment are still public concern. Therefore, the detection of predictive biomarkers at an early stage for prognostic classification and risk stratification is pivotal for CRC patients to make an individualized therapeutic plan and improve treatment response.

Focal adhesion kinase (FAK), also known as protein tyrosine kinase 2, is a non-receptor protein tyrosine kinase that is encoded by the protein tyrosine kinase2 (*PTK2*) gene (Chuang et al., 2022). It plays an important role in the progression of cell regulation, including cell adhesion, proliferation, migration, survival, angiogenesis, as well as the establishment of tumor microenvironment (TME) (Chuang et al., 2022). The overexpression and/or activation of FAK has been detected in numerous cancers, such as breast cancers, hepatocellular carcinoma, head and neck cancer, ovarian cancer and lung cancer (Mohanty et al., 2020). Besides, it is correlated with poor prognosis for cancer patients (Chen et al., 2008; Guo et al., 2019). FAK is auto-phosphorylated at Tyr-397, and the activated FAK initiates downstream signal transduction and forms interactions with intracellular proteins to stimulate migration and invasion in tumor cells (Huang et al., 2020). It was found that EGF/EGFR can phosphorylate FAK and induce epithelial-mesenchymal transition (EMT) which is considered as an important step in tumor invasion and metastasis (ee et al., 2008). Studies have demonstrated that p-FAK is positively correlated with the TNM stage in lung squamous cell carcinoma and cell differentiation in esophageal carcinomas (Murata et al., 2008; Han et al., 2013). These findings suggested that p-FAK may play a significant role in tumor formation and metastasis. Enhanced expression of p-FAK was also detected in the cytoplasm in CRC lesions and metastases tissues, and was associated with shorter disease-specific survival (Albasri et al., 2014). However, Theocharis et al. (2003) reported that FAK could not serve as a prognostic marker in CRC. So far, few relevant reports studied the role of p-FAK in CRC and were limited by small size samples. Therefore, the prognostic value of p-FAK in CRC is urgently needed to be explored in a large cohort.

In the present study, we investigated the expression of the phosphorylated FAK (p-FAK) on tumor tissues from CRC patients and adjacent normal tissues using an immunohistochemical examination on tissue microarrays (TMAs). We also evaluated the association between p-FAK expression and clinical characteristics among CRC patients, in the aim to delineate the clinical significance of p-FAK as a potential predictive biomarker for CRC progression and prognosis stratification.

## Materials and methods

### Genomic data mining

Data was obtained from the Cancer Genome Atlas (TCGA) data portal (https://www.cancer.gov/about-nci/organization/ccg/research/structural-enomics/tcga), including mRNA expression profiles and corresponding clinical information for CRC patients. *PTK2* mRNA expression in normal and tumor tissues were screened out under R 4.1.0 statistic environment (http://www.r-project.org/).

### Patients and specimen sources

This research was approved by the Ethic Committee of Changhai Hospital, Naval Medical University (Shanghai, China). A written informed consent was provided for all patients, according to the Declaration of Helsinki. During the period from January 2002 to December 2021, a total of 908 CRC patients who underwent primary resection were included in this study. 908 tumor specimens and 37 para-carcinoma tissues were collected from the surgery and tumor specimens were all pathologically diagnosed as CRC. In addition, 37 normal tissues, 33 adenoma specimens and 17 CRC liver metastasis specimens were also collected in the present study. The specimens were fixed in 4% paraformaldehyde and embedded in paraffin. Patient demographic parameters, clinical and histological characteristics were shown in Table 1, including age, sex, disease location, TNM stage based on AJCC 6th and 7th edition, tumor differentiation grade, number of resected lymph nodes, adjuvant chemotherapy, serum CEA, and serum CA 199 level. All the missing values were excluded for this study.

**TABLE 1:** Characteristics of patients with CRC.

Characteristics	Total (*n* = 908)	p-FAK expression		*p* value
		Low (IHC score <110, *n* = 463)	High (IHC score ≥110, *n* = 445)	
Age, mean ± SD (years)	60.61 ± 12.33	60.31 ± 11.95	60.93 ± 12.71	0.453
Sex, n (%)				
Male	546 (60.1%)	256 (55.3%)	290 (65.2%)	0.002
Female	362 (39.9%)	207 (44.7%)	155 (34.8%)	
Disease location, n (%)				
Rectal	475 (52.3%)	244 (52.7%)	231 (51.9%)	0.812
Colon	433 (47.7%)	219 (47.3%)	214 (48.1%)	
TNM stage, n (%)				
I	138 (15.2%)	71 (15.3%)	67 (15.1%)	0.700
II	461 (50.8%)	229 (49.5%)	232 (52.1%)	
III	309 (34.0%)	163 (35.2%)	146 (32.8%)	
Differentiation grade, n (%)				
Well	12 (1.3%)	3 (0.6%)	9 (2.0%)	0.004
Moderate	773 (85.1%)	411 (88.8%)	362 (81.3%)	
Poor	123 (13.5%)	49 (10.6%)	74 (16.6%)	
Adjuvant chemotherapy, n (%)				
No	137 (15.1%)	63 (13.6%)	74 (16.6%)	0.203
Yes	771 (84.9%)	400 (86.4%)	371 (83.4%)	
Resected lymph nodes, n (%)				
<12	200 (22.0%)	114 (24.6%)	86 (19.3%)	0.054
≥12	708 (78.0%)	349 (75.4%)	359 (80.7%)	
Serum CEA, n (%)				
<5 ng/ml	640 (70.5%)	333 (71.9%)	307 (69%)	0.333
≥5 ng/ml	268 (29.5%)	130 (28.1%)	138 (31%)	
Serum CA199, n (%)				
<37 U/ml	795 (87.6%)	410 (88.6%)	385 (86.5%)	0.368
≥37 U/ml	112 (12.3%)	52 (11.2%)	60 (13.5%)	

### Immunohistochemistry

Tissue microarrays (TMAs) containing the formalin-fixed paraffin-embedded specimens were constructed by Outdo Biotech (Shanghai, China). The TMA samples were cut into sections with 3 μm thickness and then subjected to deparaffinization. Antigen retrieval was performed in boiled 10 mM citrate buffer (pH 6.0) for 10 min. The sections were incubated with primary Phospho-FAK (Tyr397) antibody (Cell Signaling Technology, United States) at 4°C overnight and then incubated with EnVisionþDual Link System-HRP (Dako, United States). Staining was visualized using 3,3′-diaminobenzidine (Sigma, Saint Louis, MO, FAST 3,3′-diaminobenzidine).

### Quantitative evaluation of immunostaining

Stained TMA slides were observed under bright-field microscopy at a resolution of ×40 and scanned by Aperio AT2 (Leica Biosystems). The expression level of p-FAK was quantitated using an H-score as previously described (Detre et al., 1995). In brief, p-FAK staining in each fixed field was assessed by two independent observers and was scored by multiplying the staining intensity (0, negative; 1+, weak; 2+, moderate; and 3+, strong). Interobserver differences were calculated and averaged, and a third observer resolved the difference when the discrepancy exceeded 20%.

### Follow-up and survival analysis

Follow-up information for all the patients was collected, following a standard procedure as previously described (Chang et al., 2014). The primary outcomes were overall survival (OS) and disease-free survival (DFS) measured in months. DFS was defined from the date of undergoing initial surgery to the date of the first disease relapse or death. An optimal cutoff value of p-FAK IHC scores was used to divide the patients into high and low expression groups.

### Statistical analysis

All statistical analyses and charting were performed using SPSS 18.0 (SPSS Inc., Chicago, IL, United States) and GraphPad Prism 8.0 software (San Diego, United States). Two-tailed unpaired Student’s test and chi-squared tests were used for comparisons of clinical characteristics between two groups. OS and DFS were estimated with Kaplan-Meier method. Differences between subgroups were calculated and presented as hazard ratios (HR) with normal estimated 95% confidence intervals (CIs) using log-rank test. The effect of individual prognostic factors in the univariate and multivariate analyses was assessed with the Cox proportional hazards model. For these results, continuous values are presented as mean ± SD. All statistical tests were performed two-sided, and a value of *p* < 0.05 were considered as statistically significant.

## Results

### Phosphorylated focal adhesion kinase expression level was elevated in colorectal cancer

**FIGURE 1:**
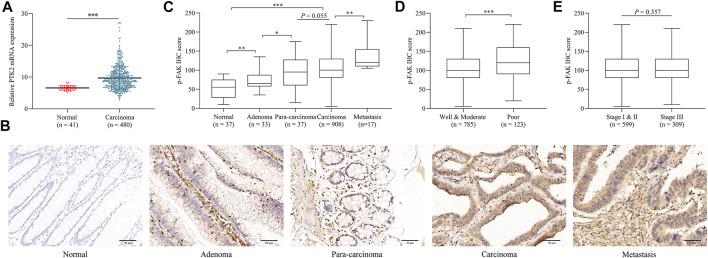
p-FAK expression in CRC. **(A)** Bioinformatics analyses of *PTK2* mRNA expression in normal tissue and CRC. **(B)** Representative images of IHC. Scalebar, 50 μm. **(C)** Comparison of IHC H-score among different colorectal pathological tissues. **(D)** Comparison of IHC H-score between different differentiation grades. **(E)** Comparison of IHC H-score between different TNM stages. The error bar represents the mean ± SD. *p < 0.05, **p < 0.01, ***p < 0.001.

We initially assessed the mRNA expression of *PTK2* in normal tissue and CRC tissue using publicly available microarray datasets. It was observed that the mRNA expression level of *PTK2* in CRC is significantly higher than in normal tissues (*p* < 0.001; Figure 1A). Then we investigated the expression pattern of p-FAK by IHC examination in normal tissue, adenoma, para-carcinoma, carcinoma, and metastasis specimens obtained from surgery. p-FAK staining was mainly distributed in the cytoplasm of epithelial cells (Figure 1B). Quantitative examination of IHC revealed that p-FAK expression level is significantly higher in carcinoma and metastasis specimens than in normal and adenoma tissues (*p* < 0.001; Figure 1C). Furthermore, we found that p-FAK IHC scores in patients with poor differentiation was significantly higher than in patients with well and moderate differentiation (*p* < 0.001; Figure 1D). There was no significant difference between stage I and II and stage III CRC patients (*p* > 0.05; Figure 1E). The results indicated an important role of p-FAK in CRC progression.

### Associations between phosphorylated focal adhesion kinase expression and clinical characteristics in colorectal cancer patients

A total of 908 patients clinically and pathologically diagnosed as CRC were divided into two group with the optimal cutoff value (IHC score = 110) using the maxstat R package: low expression group (IHC score <110, *n* = 463) and high expression group (IHC score ≥110, *n* = 445). Median follow-up for all patients was 47.9 months (5–117). Baseline characteristics of CRC patients and the comparisons between groups were shown in Table 1. There was no significant difference found in age, location, TNM stage (AJCC 6th and 7th), postoperative adjuvant chemotherapy, number of resected lymph nodes, serum CEA level and serum CA199 level between the groups (*p* > 0.05). The expression level of p-FAK was associated with differentiation grade (*p* < 0.01) and sex (*p* < 0.01). These results indicated that p-FAK may contributed to the aggressiveness of CRC.

### Phosphorylated focal adhesion kinase expression predicted the prognosis of colorectal cancer as an independent factor

**FIGURE 2:**
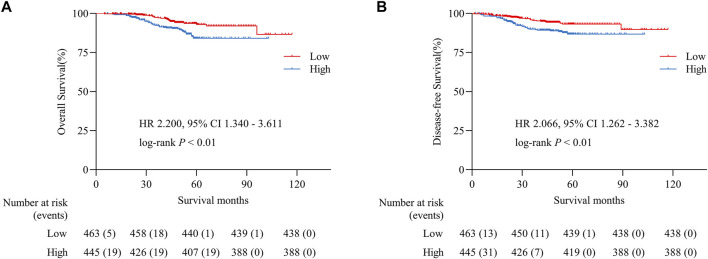
Overall survival **(A)** and disease-free survival **(B)** in CRC patients. HR, hazard ratio.

We constructed Kaplan-Meier survival curves and determined the hazard ratios (HRs) using the Cox proportional hazards model. Differences of OS and DFS between low and high groups were compared using the log-rank test. 25 deaths occurred overall in 463 patients in the low group and 44 occurred in 445 patients in the high group (unadjusted HR 2.200, 95% CI 2.340–3.611, *p* < 0.01; Figure 2A). 20 deaths attributed to recurrence occurred in the low group compared with 38 in the high group (unadjusted HR 2.066, 95% CI 1.262–3.382, *p* < 0.01; Figure 2B). It revealed that high expression level of p-FAK was associated with shorter OS and DFS in CRC patients. Having adjusted for other prognostic factors, including age, sex, location, TNM stage, differentiation grade, adjuvant chemotherapy, resected lymph nodes, serum CEA level and serum CA199 level, the expression level of p-FAK remained an independent risk factor for OS with a HR of 2.090 (95% CI, 1.265–3.452; *p* < 0.01) and for DFS with a HR of 2.004 (95% CI, 1.214–3.308; *p* < 0.01) in a multivariable analysis (Table 2).

**TABLE 2:** Multivariable analysis of prognostic factors in overall survival and disease-specific survival.

	Overall survival		Disease-specific survival	
	Hazard ratio (95% CI)	p value	Hazard ratio (95% CI)	p value
p-FAK expression		0.004		0.007
Low	1.00^a^		1.00^a^	
High	2.090 (1.265–3.452)		2.004 (1.214–3.308)	
Age (years)		0.595		0.566
<60	1.00^a^		1.00^a^	
≥60	0.877 (0.540–1.425)		0.868 (0.534–1.410)	
Sex		0.775		0.908
Male	1.00^a^		1.00^a^	
Female	1.075 (0.654–1.766)		1.03 (0.628–1.687)	
Disease location		0.288		0.266
Rectal	1.00^a^		1.00^a^	
Colon	0.765 (0.466–1.255)		0.755 (0.461–1.238)	
TNM stage		0.558		0.499
I and II	1.00^a^		1.00^a^	
III	0.859 (0.518–1.426)		0.839 (0.505–1.395)	
Differentiation grade		0.436		0.652
Well and Moderate	1.00^a^		1.00^a^	
Poor	0.767 (0.393–1.496)		0.858 (0.442–1.667)	
Adjuvant chemotherapy		0.717		0.662
No	1.00^a^		1.00^a^	
Yes	0.873 (0.419–1.820)		0.849 (0.407–1.771)	
Resected lymph nodes		0.076		0.204
<12	1.00^a^		1.00^a^	
≥12	0.569 (0.306–1.060)		0.672 (0.364–1.240)	
Serum CEA (ng/ml)		0.109		0.081
<5	1.00^a^		1.00^a^	
≥5	0.659 (0.395–1.097)		0.635 (0.381–1.058)	
Serum CA199 (U/ml)		0.819		0.785
<37	1.00^a^		1.00^a^	
≥37	1.090 (0.395–1.097)		1.108 (0.529–2.323)	

a Reference group.

### Low phosphorylated focal adhesion kinase expression predicted a better prognosis in early-stage colorectal cancer patients

**FIGURE 3:**
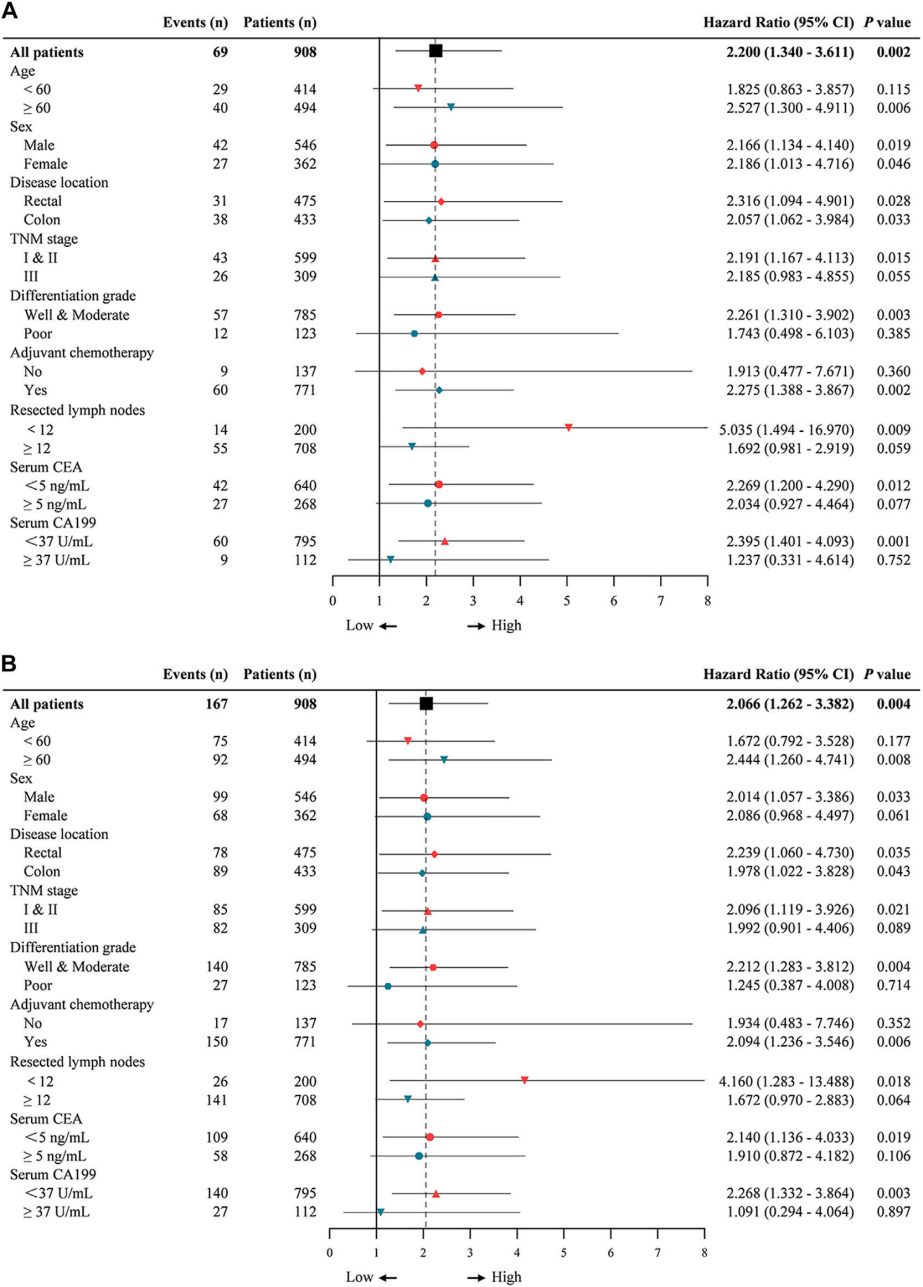
Univariable subgroup analyses of overall survival **(A)** and disease-free survival **(B)** in CRC patients. The dotted line shows the hazard ratio for all patients.

Then we estimated interactions between p-FAK expression and age, sex, location, TNM stage, differentiation grade, adjuvant chemotherapy, resected lymph nodes, serum CEA level and serum CA199 level in a post-hoc subgroup analysis. As shown in Figure 3, subgroups of age ≥60 years, rectal, colon, TNM stage I and II, well and moderate differentiation grade, with adjuvant chemotherapy, resected lymph nodes <12, serum CEA <5 ng/ml and serum CA199 < 37 U/ml were significant for either OS and DFS (*p* < 0.05), and the subgroup of female was only significant for OS (*p* < 0.05). Low p-FAK expression was associated with a good prognosis in early-stage CRC patients and patients with low-risk factors compared with high p-FAK expression group.

### Phosphorylated focal adhesion kinase expression predicted response to adjuvant chemotherapy in colorectal cancer patients

**FIGURE 4:**
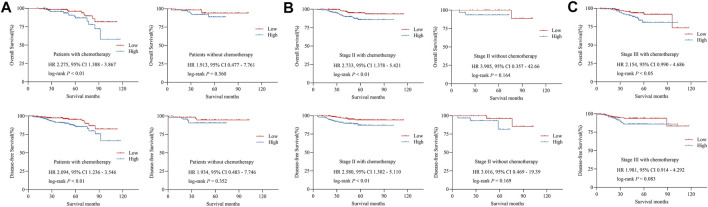
**(A)** Overall survival and disease-free survival in patients with and without adjuvant chemotherapy. **(B)** Overall survival and disease-free survival in stage II CRC patients with and without adjuvant chemotherapy. **(C)** Overall survival and disease-free survival in stage III CRC patients with adjuvant chemotherapy. HR, hazard ratio.

In our cohort, all stage III patients and stage II patients who diagnosed with high-risk factors received adjuvant chemotherapy, according to the current guidelines of CRC. We attempted to identify the relationship between p-FAK expression and survival outcomes among the patients with adjuvant chemotherapy. As shown in Table 2 and Figure 4A, high p-FAK expression was related to a shorter OS (*p* < 0.01) and DFS (*p* < 0.01) in CRC patients who achieved adjuvant chemotherapy. No significance was observed in those patients without chemotherapy (*p* > 0.05). In those patients who achieved adjuvant chemotherapy, high expression level of p-FAK indicated both shorter OS (*p* < 0.01) and DFS (*p* < 0.01) for patients with stage II CRC (Figure 4B), and only shorter OS (*p* < 0.05) for stage III CRC patients (Figure 4C). There was no significant difference in stage II patients without adjuvant chemotherapy (*p* > 0.05, Figure 4B). Then we performed the interaction analysis of p-FAK expression in patients with or without chemotherapy. It was found that there was no interaction between p-FAK expression and adjuvant chemotherapy on the outcomes of CRC patients (*p* > 0.05, Table 3). It demonstrated that p-FAK expression may predict the chemotherapeutic response of CRC patients, especially for early-stage patients.

**TABLE 3:** Effect of p-FAK expression on the prediction of chemotherapy response in CRC patients.

	Overall survival					Disease-specific survival				
	Low group total (events)	High group total (events)	HR (95%CI)	*p* value	*p* value for interaction	Low group (Events)	High group total (events)	HR (95%CI)	*p* value	*p* value for interaction
Adjuvant chemotherapy					0.963					0.925
Without	63 (3)	74 (6)	1.913 (0.477–7.761)	0.360		63 (6)	74 (11)	1.934 (0.483–7.746)	0.352	
With	400 (22)	371 (38)	2.275 (1.388–3.867)	<0.01		400 (70)	371 (80)	2.094 (1.236–3.546)	<0.01	

## Discussion

Previous studies have identified the relationship between FAK activation and accelerated cancer growth and invasion in various human cancers (Mohanty et al., 2020). Overexpression of p-FAK was intimately associated with TNM classification, lymph node metastasis and distant metastasis in some malignancies. In few research performed in small cohorts, enhanced expression of FAK and p-FAK was noted in CRC (Yu et al., 2006; Garouniatis et al., 2013; Albasri et al., 2014), but the correlations between raised FAK expression and tumor stage, Ki-67 positivity or survival in CRC have been inconsistent in Theocharis’s study (Theocharis et al., 2003). Therefore, we conduct the current study with the aim of identifying the role of p-FAK in CRC. We confirmed that *PTK2* mRNA was high expressed in CRC tissues using publicly available expression profiling data. We further investigate p-FAK expression pattern by IHC examination and the result was consistent with that from *PTK2* mRNA. Strong straining was also observed in metastasis tissues. Besides, high expression of p-FAK was associated with poor differentiation. From these data, it can be inferred that p-FAK may contribute to CRC progression. Recent studies *in vitro* have revealed the FAK involvement in the process of epithelial-to-mesenchymal transition (EMT) which capacitates epithelial tumor cells to acquire invasive properties and metastatic growth characteristics (Yoon et al., 2015). Activated FAK was important in regulating a decrease of epithelial markers like E-cadherin and an increase of mesenchymal markers required for EMT (Chuang et al., 2022). p-FAK was associated with Src-regulated E-cadherin expression in colon cancer cells and its overexpression promoted cell invasion and metastasis (Hauck et al., 2002).

In univariate Cox analysis, we found that high expression of p-FAK is correlated with worse prognosis in CRC patients. Traditionally, clinicopathological features are considered having influence on the survival of CRC patients, including TNM stage, histological type, number of resected lymph nodes, and tumor markers of CEA and CA199. These factors are used as predictors of recurrence in CRC clinically, but relying solely on them are insufficient for accurate prediction of early recurrence. We performed a multivariate Cox proportional hazards model to balance the effect of other factors, and the results revealed that the outcomes of patients with high FAK expression were poor, which indicated that p-FAK is an independent prognostic factor in the general population with CRC. These results are consistent with precious studies performed by Liu et al. (2013), Albasri et al. (2014). The promising prognostic role of p-FAK in CRC is consistent with lung squamous cell carcinoma and hepatocellular carcinoma (Han et al., 2013; Guo et al., 2019).

Moreover, we assessed whether other predictors versus p-FAK expression were related to the treatment outcomes using a post-hoc subgroup analysis. Expression level of p-FAK is significantly associated with OS and DFS in TNM stage I and II, well and moderate differentiation grade, resected lymph nodes <12, serum CEA <5 ng/ml, and serum CA199 < 37 U/ml subgroups, of which were predictors of a beneficial outcome for CRC. It suggested that p-FAK expression might be a supplementary IHC biomarker for CRC prognosis, especially useful in early-stage patients with traditionally favorable predictors. Though our study performed in a large cohort, some information and factors including MSI, extramural venous invasion, and KRAS or BRAF mutation were not available, which need a larger prospective cohort in future studies.

At present, surgical resection is the only potentially curative treatment for CRC. Adjuvant chemotherapy is used to eradicate micro-metastatic disease for those high-risk stage II and stage III patients with the aim of improving survival. However, a proportion of these patients received limited benefits from chemotherapy and the outcomes are often unpredictable. Thus, our study further focused on the potential role of p-FAK expression status in predicting adjuvant chemotherapy response. We found that high p-FAK expression was related to a poor outcome in CRC patients with chemotherapy and no difference was noted in patients without chemotherapy. The difference was significantly observed in stage II CRC patients that was consistent with the results above. Our findings suggested that p-FAK could be a valuable biomarker in evaluating patients’ response to chemotherapy.

FAK signaling pathway is a key role in driving cell functions regulated by integrins and growth receptor factors (Mohanty et al., 2020; Chuang et al., 2022). Overexpression of FAK and p-FAK in numerous cancers indicates that targeting FAK might be effective in suppressing biological capacities required for tumorigenesis which makes it an attractive drug target for anti-cancer therapy (Sharma et al., 2017; Pomella et al., 2022). The inhibitors blunt FAK Try-397 auto-phosphorylation and affects downstream signaling pathways including PI3K/AKT, STAT3 and JNK in some cancer cells, and further influence cell viability, proliferation, migration, and immunosuppressive TME (Zhang et al., 2014; Hirt et al., 2018). Experiments *in vivo* indicated that tumor growth and metastasis were reduced by FAK inhibitor in breast cancer mouse model, human glioblastoma xenografted model and adenocarcinoma xenograft model (Brown et al., 2018; Hirt et al., 2018; Tiede et al., 2018). In addition, FAK is known to mediate resistance to chemotherapeutics. It is reported that activated FAK regulated Y-box binding protein 1 to induce paclitaxel resistance in ovarian cancer while FAK inhibitor could reduce the resistance (Kang et al., 2013). Currently, a big effort in several Phase I and II clinical trials have been activated to evaluate the efficacy of FAK inhibitors in cancer treatment as single agents or in combination with chemotherapy (Pomella et al., 2022). FAK inhibitors may become effective in reversing insensitive chemotherapy response for early-stage CRC patients with high p-FAK expression that remains future studies.

In conclusion, the current study identified the important role of p-FAK as a potential predictive biomarker for prognosis and chemotherapy response in CRC patients, especially in early-stage patients. It suggested that high p-FAK expression is associated with unfavorable outcome and poor chemotherapeutic response in CRC. We hope our study can help carry out the early diagnosis in order to reduce disease-free progression and risk of recurrence. Furthermore, the precise biological functions and possible molecular mechanisms of p-FAK in CRC tumorigenesis and progression remain unknown. The potential of p-FAK as a therapeutic target in the antineoplastic therapy is required to be further elucidated.

## Data Availability

The original contributions presented in the study are included in the article/Supplementary Materials, further inquiries can be directed to the corresponding authors.

## References

[B1] Albasri A. Fadhil W. Scholefield J. H. Durrant L. G. Ilyas M. (2014). Nuclear expression of phosphorylated focal adhesion kinase is associated with poor prognosis in human colorectal cancer. Anticancer Res. 34 (8), 3969–3974. 25075018

[B2] Biller L. H. Schrag D. (2021). Diagnosis and treatment of metastatic colorectal cancer: A review. Jama 325 (7), 669–685. 10.1001/jama.2021.0106 33591350

[B3] Bray F. Ferlay J. Soerjomataram I. Siegel R. L. Torre L. A. Jemal A. (2018). Global cancer statistics 2018: GLOBOCAN estimates of incidence and mortality worldwide for 36 cancers in 185 countries. Ca. Cancer J. Clin. 68 (6), 394–424. 10.3322/caac.21492 30207593

[B4] Brown N. F. Williams M. Arkenau H. T. Fleming R. A. Tolson J. Yan L. (2018). A study of the focal adhesion kinase inhibitor GSK2256098 in patients with recurrent glioblastoma with evaluation of tumor penetration of [11C]GSK2256098. Neuro. Oncol. 20 (12), 1634–1642. 10.1093/neuonc/noy078 29788497 PMC6231205

[B5] Buccafusca G. Proserpio I. Tralongo A. C. Rametta Giuliano S. Tralongo P. (2019). Early colorectal cancer: Diagnosis, treatment and survivorship care. Crit. Rev. Oncol. Hematol. 136, 20–30. 10.1016/j.critrevonc.2019.01.023 30878125

[B6] Chang W. Gao X. Han Y. Du Y. Liu Q. Wang L. (2014). Gene expression profiling-derived immunohistochemistry signature with high prognostic value in colorectal carcinoma. Gut 63 (9), 1457–1467. 10.1136/gutjnl-2013-305475 24173294

[B7] Chen S. Y. Moroi Y. Urabe K. Takeuchi S. Kido M. Hayashida S. (2008). Concordant overexpression of p-FAK and p-ERK1/2 in extramammary Paget's disease. Arch. Dermatol. Res. 300 (4), 195–201. 10.1007/s00403-008-0829-2 18210145

[B8] Chuang H. H. Zhen Y. Y. Tsai Y. C. Chuang C. H. Hsiao M. Huang M. S. (2022). FAK in cancer: From mechanisms to therapeutic strategies. Int. J. Mol. Sci. 23 (3), 1726. 10.3390/ijms23031726 35163650 PMC8836199

[B9] Dekker E. Tanis P. J. Vleugels J. L. A. Kasi P. M. Wallace M. B. (2019). Colorectal cancer. Lancet 394 (10207), 1467–1480. 10.1016/S0140-6736(19)32319-0 31631858

[B10] Detre S. Saclani Jotti G. Dowsett M. (1995). A "quickscore" method for immunohistochemical semiquantitation: Validation for oestrogen receptor in breast carcinomas. J. Clin. Pathol. 48 (9), 876–878. 10.1136/jcp.48.9.876 7490328 PMC502883

[B11] ee M. Y. Chou C. Y. Tang M. J. Shen M. R. (2008). Epithelial-mesenchymal transition in cervical cancer: Correlation with tumor progression, epidermal growth factor receptor overexpression, and snail up-regulation. Clin. Cancer Res. 14 (15), 4743–4750. 10.1158/1078-0432.CCR-08-0234 18676743

[B12] Garouniatis A. Zizi-Sermpetzoglou A. Rizos S. Kostakis A. Nikiteas N. Papavassiliou A. G. (2013). FAK, CD44v6, c-met and EGFR in colorectal cancer parameters: Tumour progression, metastasis, patient survival and receptor crosstalk. Int. J. Colorectal Dis. 28 (1), 9–18. 10.1007/s00384-012-1520-9 22733437

[B13] Guo P. He Y. Chen L. Qi L. Liu D. Chen Z. (2019). Cytosolic phospholipase A2α modulates cell-matrix adhesion via the FAK/paxillin pathway in hepatocellular carcinoma. Cancer Biol. Med. 16 (2), 377–390. 10.20892/j.issn.2095-3941.2018.0386 31516757 PMC6713643

[B14] Han X. Xue L. Zhou L. Gong L. Zhu S. Yao L. (2013). The role of PTPN13 in invasion and metastasis of lung squamous cell carcinoma. Exp. Mol. Pathol. 95 (3), 270–275. 10.1016/j.yexmp.2013.07.008 23906871

[B15] Hauck C. R. Hsia D. A. Schlaepfer D. D. (2002). The focal adhesion kinase--a regulator of cell migration and invasion. IUBMB Life 53 (2), 115–119. 10.1080/15216540211470 12049193

[B16] Hirt U. A. Waizenegger I. C. Schweifer N. Haslinger C. Gerlach D. Braunger J. (2018). Efficacy of the highly selective focal adhesion kinase inhibitor BI 853520 in adenocarcinoma xenograft models is linked to a mesenchymal tumor phenotype. Oncogenesis 7 (2), 21. 10.1038/s41389-018-0032-z 29472531 PMC5833389

[B17] Huang K. Gao N. Bian D. Zhai Q. Yang P. Li M. (2020). Correlation between FAK and EGF-induced EMT in colorectal cancer cells. J. Oncol. 2020, 5428920. 10.1155/2020/5428920 32148496 PMC7048944

[B18] Kang Y. Hu W. Ivan C. Dalton H. J. Miyake T. Pecot C. V. (2013). Role of focal adhesion kinase in regulating YB-1-mediated paclitaxel resistance in ovarian cancer. J. Natl. Cancer Inst. 105 (19), 1485–1495. 10.1093/jnci/djt210 24062525 PMC3787907

[B19] Liu S. Q. Su Y. J. Qin M. B. Mao Y. B. Huang J. A. Tang G. D. (2013). Sphingosine kinase 1 promotes tumor progression and confers malignancy phenotypes of colon cancer by regulating the focal adhesion kinase pathway and adhesion molecules. Int. J. Oncol. 42 (2), 617–626. 10.3892/ijo.2012.1733 23232649

[B20] Mohanty A. Pharaon R. R. Nam A. Salgia S. Kulkarni P. Massarelli E. (2020). FAK-Targeted and combination therapies for the treatment of cancer: An overview of phase I and II clinical trials. Expert Opin. Investig. Drugs 29 (4), 399–409. 10.1080/13543784.2020.1740680 32178538

[B21] Murata T. Naomoto Y. Yamatsuji T. Okawa T. Shirakawa Y. Gunduz M. (2008). Localization of FAK is related with colorectal carcinogenesis. Int. J. Oncol. 32 (4), 791–796. 18360706

[B22] Nagtegaal I. D. Quirke P. Schmoll H. J. (2011). Has the new TNM classification for colorectal cancer improved care? Nat. Rev. Clin. Oncol. 9 (2), 119–123. 10.1038/nrclinonc.2011.157 22009076

[B23] Pomella S. Cassandri M. Braghini M. R. Marampon F. Alisi A. Rota R. (2022). New insights on the nuclear functions and targeting of FAK in cancer. Int. J. Mol. Sci. 23 (4), 1998. 10.3390/ijms23041998 35216114 PMC8874710

[B24] Sharma P. Hu-Lieskovan S. Wargo J. A. Ribas A. (2017). Primary, adaptive, and acquired resistance to cancer immunotherapy. Cell. 168 (4), 707–723. 10.1016/j.cell.2017.01.017 28187290 PMC5391692

[B25] Theocharis S. E. Kouraklis G. P. Kakisis J. D. Kanelli H. G. Apostolakou F. E. Karatzas G. M. (2003). Focal adhesion kinase expression is not a prognostic predictor in colon adenocarcinoma patients. Eur. J. Surg. Oncol. 29 (7), 571–574. 10.1016/s0748-7983(03)00120-3 12943621

[B26] Tiede S. Meyer-Schaller N. Kalathur R. K. R. Ivanek R. Fagiani E. Schmassmann P. (2018). The FAK inhibitor BI 853520 exerts anti-tumor effects in breast cancer. Oncogenesis 7 (9), 73. 10.1038/s41389-018-0083-1 30237500 PMC6148276

[B27] Xia C. Dong X. Li H. Cao M. Sun D. He S. (2022). Cancer statistics in China and United States, 2022: Profiles, trends, and determinants. Chin. Med. J. 135 (5), 584–590. 10.1097/CM9.0000000000002108 35143424 PMC8920425

[B28] Yoon H. Dehart J. P. Murphy J. M. Lim S. T. (2015). Understanding the roles of FAK in cancer: Inhibitors, genetic models, and new insights. J. Histochem. Cytochem. 63 (2), 114–128. 10.1369/0022155414561498 25380750 PMC4305513

[B29] Yu H. G. Tong S. L. Ding Y. M. Ding J. Fang X. M. Zhang X. F. (2006). Enhanced expression of cholecystokinin-2 receptor promotes the progression of colon cancer through activation of focal adhesion kinase. Int. J. Cancer 119 (12), 2724–2732. 10.1002/ijc.22207 16998832

[B30] Zhang J. He D. H. Zajac-Kaye M. Hochwald S. N. (2014). A small molecule FAK kinase inhibitor, GSK2256098, inhibits growth and survival of pancreatic ductal adenocarcinoma cells. Cell. Cycle 13 (19), 3143–3149. 10.4161/15384101.2014.949550 25486573 PMC4615113

